# Distribution and Clinical Significance of IL-17A in Tumor-Infiltrating Lymphocytes of Non-Small Cell Lung Cancer Patients

**DOI:** 10.3389/pore.2022.1610384

**Published:** 2022-05-18

**Authors:** Rui Xu, Xing Ke, Wenwen Shang, Shuna Liu, Xin Fu, Ting Wang, Shuxian Jin

**Affiliations:** ^1^ Department of Laboratory Medicine, The First Affiliated Hospital of Nanjing Medical University, Nanjing, China; ^2^ Branch of National Clinical Research Center for Laboratory Medicine, Nanjing, China; ^3^ Department of Clinical Laboratory, Xinhua Hospital, Shanghai Jiao Tong University School of Medicine, Shanghai, China; ^4^ Department of Respiratory and Critical Care Medicine, The First Affiliated Hospital of Nanjing Medical University, Nanjing, China

**Keywords:** NSCLC, TILs, Th17 cells, Tc17 cells, γδT cells, IL-17

## Abstract

**Objective:** To investigate the distribution of IL-17A and its clinical significance in tumor infiltrating lymphocytes (TILs) of patients with non-small cell lung cancer (NSCLC).

**Methods:** Expression level of IL-17A in TILs of 3 paired NSCLC and paracancerous specimens was measured by qRT-PCR. The distribution of IL-17A in immune cell subsets of 15 paired NSCLC and paracancerous specimens was examined by flow cytometry. The correlation between IL-17A and clinical features of NSCLC was identified.

**Results:** IL-17A was significantly upregulated in TILs of NSCLC specimens than those of paracancerous ones (*p* < 0.0001). Meanwhile, T helper 17 cells (Th17 cells, *p* < 0.001), IL-17-secreting CD8^+^ T cells (Tc17 cells, *p* < 0.001) and IL-17-producing cells (γδT17 cells, *p* < 0.0001) were significantly abundant in TILs of NSCLC specimens than those of controls, and the higher abundance of the latter was much pronounced than that of the former two. Moreover, γδT17 cells in TILs were significantly correlated with lymphatic metastasis and CYFRA 21-1 level of NSCLC patients (*p* < 0.05).

**Conclusion:** Tumor infiltrated γδT cells are the main source of IL-17 in early-stage NSCLC, and IL-17 may be a vital regulator involved in the development of NSCLC.

## Introduction

Lung cancer has been the leading cancer in the world, accounting for about 13% of all cancer cases. More seriously, its cancer mortality ranks first, leading to more than 1.4 million deaths per year worldwide. Non-small cell lung cancer (NSCLC) is the main subtype of lung cancer, covering 80–85% of lung cancer cases. NSCLC patients are poorly responsive to conventional therapeutic strategies like surgery, chemotherapy and radiotherapy, with the 5-year survival of lower than 15% [1]. The pathogenesis of NSCLC is very complicated. In recent years, the correlation between tumor and inflammation has been highlighted, and inflammation is closely linked with tumor metastasis, neovascularization and tumor immunity [2].

IL-17 is a class of T cell-derived proinflammatory cytokines, which is mainly produced by CD4^+^ T cells (Th17), CD8^+^ T cells (Tc17) and γδT (γδT17) cells. The IL-17 family consists of six members, including IL-17A, IL-17B, IL-17C, IL-17D, IL-17E (IL-25) and IL-17F. IL-17A is often referred to IL-17 due to the highest homology. It is widely involved in infectious diseases, autoimmune diseases and tumors via upregulating intercellular adhesion molecule-1 (ICAM-1), mediating the infiltration of inflammatory cells and T lymphocytes, and producing antibodies with the synergistic assistance of cytokines. Previous studies have shown that IL-17A can promote tumor immunosuppression and induce immune escape in colorectal cancer. IL-17A aggravates the development of gastric cancer, leading to a poor prognosis [3, 4]. In the present study, we aim to investigate the role of IL-17A in the development of NSCLC by exploring the distribution of IL-17A in TILs of NSCLC and its correlation with clinicopathological features of NSCLC patients.^1^

## Materials and Methods

### Subjects and Tissue Specimens

The study was authorized by the Ethics Committee of the First Affiliated Hospital of Nanjing Medical University (Nanjing, China, 2017-SRFA-064). A total of 15 NSCLC patients with the mean age of 61 (35–72) years who were treated in the First Affiliated Hospital of Nanjing Medical University between 2017 and 2018 were recruited, including 8 male and 7 female cases. Among them, there were 13 cases of lung adenocarcinoma and 2 of lung squamous cell carcinoma. All patients were excluded chronic obstructive pulmonary disease (COPD). NSCLC specimens and paracancerous ones were collected for use. According to the latest version of the Union for International Cancer Control (UICC) TNM Classification of Lung Cancer, there were 2 cases of stage I, 12 of stage II, and 1 of stage III. All recruited subjects did not have history of surgery, chemotherapy, radiotherapy and immunosuppressive therapy.

### Tumor Infiltrating Lymphocytes Separation

Fresh NSCLC and paracancerous tissues specimens were cut into small pieces and digested in RPMI-1640 medium containing 50 units/ml DNase I, 100 μg/ml hyaluronidase, 1 mg/ml Collagenase Type IV and 2 μM l-glutamine (Sigma-Aldrich) at room temperature for 2 h. Single-cell suspension was obtained after filtration using a 100-mm screen (BD Falcon, San Jose, CA, United States), and TILs were obtained from the cell suspension by the Percoll density gradient separation (GE HealthCare Life Sciences, Piscataway, NJ, United States). 30% (1.043 g/ml) and 70% (1.090 g/ml) Percoll solution were used.

### RNA Isolation and Quantitative Real-Time PCR

Total RNA was isolated from TILs using TRIzol (Life Technologies, Foster City, CA, United States), and then reversely transcribed to cDNA with Prime Script RT Master Mix (Takara, Otsu, Japan) according to the manufacturer’s instructions. Relative levels were measured using the SYBR Green Master Mix on the 7500 Real-Time PCR system (Applied Biosystems; Life Technologies, Grand Island, NY, United States). IL-17 mRNA primers were designed by primer premier 5.0 software. The primer sequences were: IL-17, 5′-ACC​AAT​CCC​AAA​AGG​TCC​TC-3′ (forward) and 5′-GGG​GAC​AGA​GTT​CAT​GTG​GT-3′ (reverse); glyceraldehyde 3-phosphate dehydrogenase (GAPDH), 5′-GAA​GGT​CGG​TGT​GAA​CGG​A-3′ (forward) and 5′-GTT​AGT​GGG​GTC​TCG​CTC​CT-3′ (reverse). Relative levels were calculated by the 2^−ΔΔCt^ method and normalized to that of GAPDH.

### Flow Cytometry

TILs were washed with phosphate-buffered saline (PBS) containing 1% fetal bovine serum (FBS). Aliquots of single cell suspensions (1×10^6^) were incubated with fluorophore-conjugated monoclonal antibodies for cell surface staining at room temperature in the dark for 20 min (Alexa Fluor 700 anti-human CD3 antibody, Alexa Fluor 750 anti-human CD45 antibody, PE anti-human CD4 antibody, FITC anti-human CD8 antibody, and/or BV421 anti-human TCRγδ antibody; all from Biolegend, San Jose, CA, United States). For intracellular staining, cells were cultured in RPMI-1640 medium containing 10% FBS, 50 ng/ml phorbol 12-myristate 13-acetate, 1 μg/ml ionomycin, and 1 μg/ml brefeldin A (Biogems, Rocky Hill, NJ, United States) by for 5 h at 37°C. Cells were then washed with PBS containing 1% FBS, fixed, permeabilized using Intracellular staining perm wash buffer kit (Biolegend, San Jose, CA, United States) according to the manufacturer’s protocol, and stained with APC anti-IL-17A antibody (Biolegend, San Jose, CA, United States). Cells without staining were used as control. Th17 cells were defined as CD3^+^CD4^+^IL-17^+^ cells. Tc17 cells were defined as CD3^+^CD8^+^IL-17^+^ cells and γδT17 cells were defined as CD3^+^TCRγδ^+^IL-17^+^ cells. Fluorescence signals were collected on a FACS Aria II (BD Biosciences, San Jose, CA, United States) and analyzed with FlowJo software (Tree Star, Ashland, OR, United States). Absolute value of positive cells in 1 × 10^6^ cells was calculated based on the positive percentage.

### Tumor Markers Detection

The levels of serum CYFRA 21-1 and CEA were detected by Roche Cobas E602 automatic electrochemiluminescence immunoanalyzer (ROCHE, Basel, Switzerland) in Department of Laboratory Medicine of the First Affiliated Hospital of Nanjing Medical University. The normal reference value range of CYFRA 21-1 is <3.3 ng/ml and that of CEA is <5 ng/ml.

### Statistical Analysis

Statistical analysis was performed using SPSS (Statistical Package for the Social Science) 20.0 statistical software (IBM Corp, Armonk, NY, United States). Pearson test was used to test whether the data come from a Gaussian distribution. Differences between the two groups were evaluated using the Student’s *t* test. The non-parametric Mann–Whitney *U* test was chosen if the sample size was too small or not fit Gaussian distribution (result of RT-PCR, the correlation between γδT17 and lymph node metastasis). All data were expressed as mean ± standard error (SEM). *p* values less than 0.05 were considered statistically significant.

## Results

### IL-17A Is Upregulated in Tumor Infiltrating Lymphocytes of Non-Small Cell Lung Cancer Specimens

**FIGURE 1:**
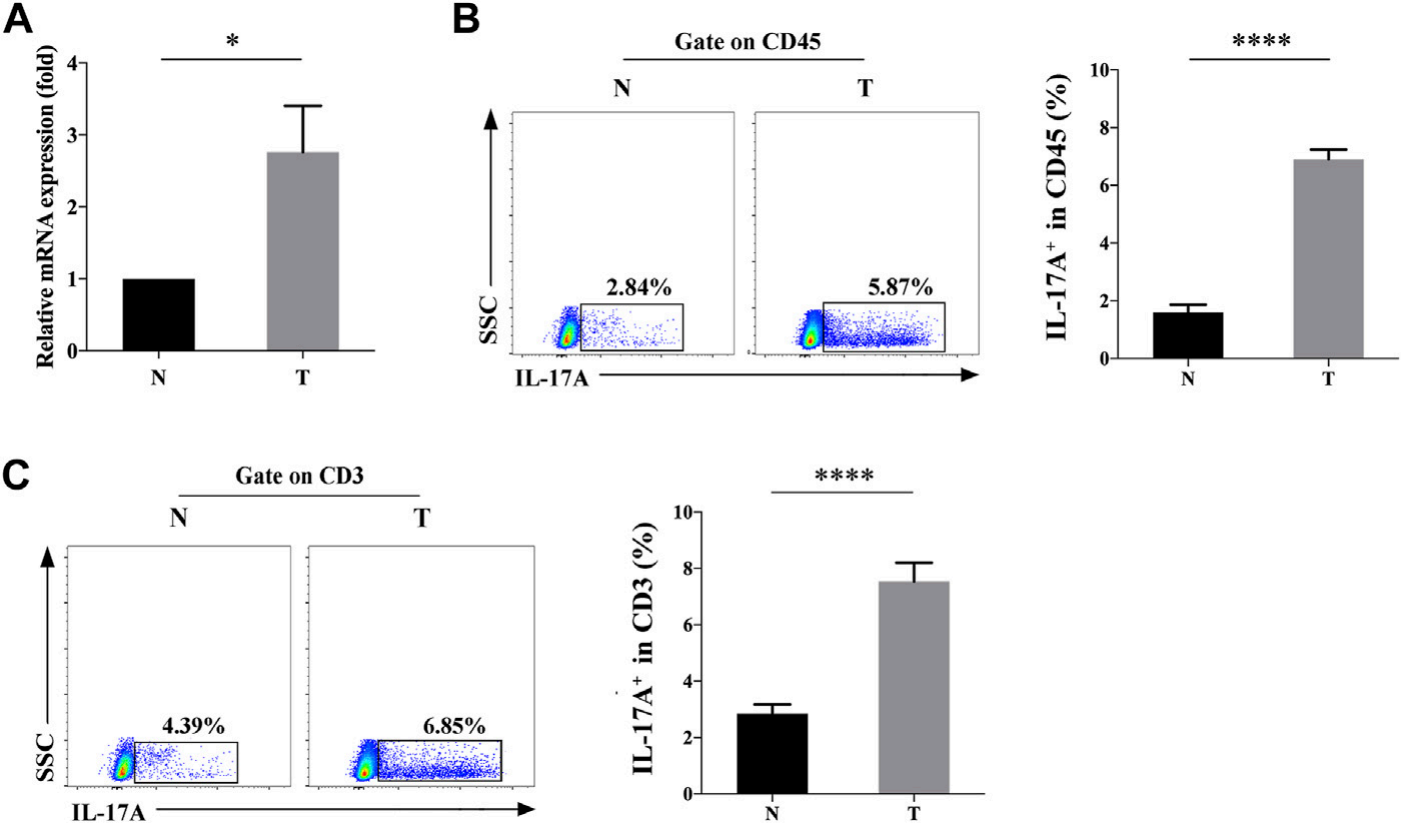
Expression level of IL-17A in TILs of NSCLC (T) and paracancerous specimens (N). **(A)** Relative levels of IL-17A in TILs of NSCLC (T, *n* = 3) and paracancerous specimens (N, *n* = 3); **(B)** The positive rate of IL-17A^+^ cells in CD45^+^ cells of NSCLC (T, *n* = 15) and paracancerous specimens (N, *n* = 15); **(C)** The positive rate of IL-17A^+^ cells in CD45^+^CD3^+^ cells of NSCLC (T, *n* = 15) and paracancerous specimens (N, *n* = 15). ****p < 0.0001.

The mRNA levels of IL-17A in TILs of NSCLC and paracancerous specimens were measured by qRT-PCR, which was significantly upregulated (2.208 ± 0.355 fold) in the former (*p* < 0.05, Figure 1A). Consistently, flow cytometry data also revealed the significantly higher positive expression rate (6.9 ± 0.34% vs. 1.60 ± 0.26%) of IL-17A in TILs of NSCLC specimens than those of controls (*p* < 0.0001, Figure 1B). To further confirm the source of IL-17, we found that both the positive percentage (7.54 ± 0.67% vs. 2.85 ± 0.33%) of CD3^+^IL-17^+^ cells were significantly higher in TILs of NSCLC specimens than those of paracancerous ones (*p* < 0.0001, Figure 1C), suggesting that CD3^+^ T cells was the main source of IL-17A.

### γδT Cells Were the Main Source of IL-17A in Non-Small Cell Lung Cancer Tissues

**FIGURE 2:**
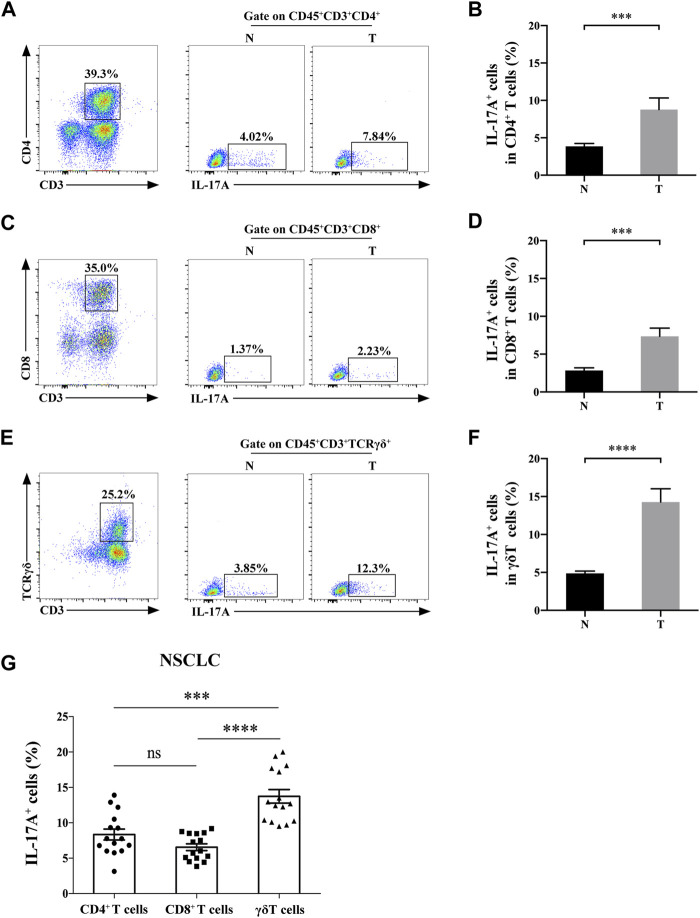
γδT cells are the main source of IL-17A in NSCLC tissues. The positive rate of IL-17A^+^ cells in CD3^+^CD4^+^ cells **(A,B)**, CD3^+^CD8^+^ cells **(C,D)** and γδT cells **(E,F)** of NSCLC (T, *n* = 15) and paracancerous specimens (N, *n* = 15); **(G)** The positive rate of IL-17A^+^ cells in Th17 cells,Tc17 cells and γδT17 cells of NSCLC (*n* = 15). ***p < 0.001; ****p < 0.0001.

We then detected the expression level of IL-17A in CD3^+^ T cell subsets. IL-17 was expressed in infiltrated CD3^+^CD4^+^ cells, CD3^+^CD8^+^ cells and γδT cells of both NSCLC and paracancerous tissues (Figures 2A–F). Moreover, the abundance of IL-17A in Th17 cells (8.77 ± 1.56% vs. 3.85 ± 0.39%), Tc17 cells (7.34 ± 1.09% vs. 2.85 ± 0.34%) and γδT17 cells (14.27 ± 1.75% vs. 4.88 ± 0.30%) isolated from NSCLC specimens was significantly higher than those isolated from paracancerous ones (all *p* < 0.0001). In addition, the positive percentage of IL-17 in γδT17 cells isolated from NSCLC specimens were significantly higher than those of Th17 and Tc17 cells in NSCLC (*p* < 0.001, *p* < 0.0001, respectively, Figure 2G). These results suggested that γδT cells were the main source of IL-17A in TILs of NSCLC.

### The Abundance of IL-17^+^ γδT Cells Is Correlated With Clinicopathological Features of Non-Small Cell Lung Cancer Patients

**FIGURE 3:**
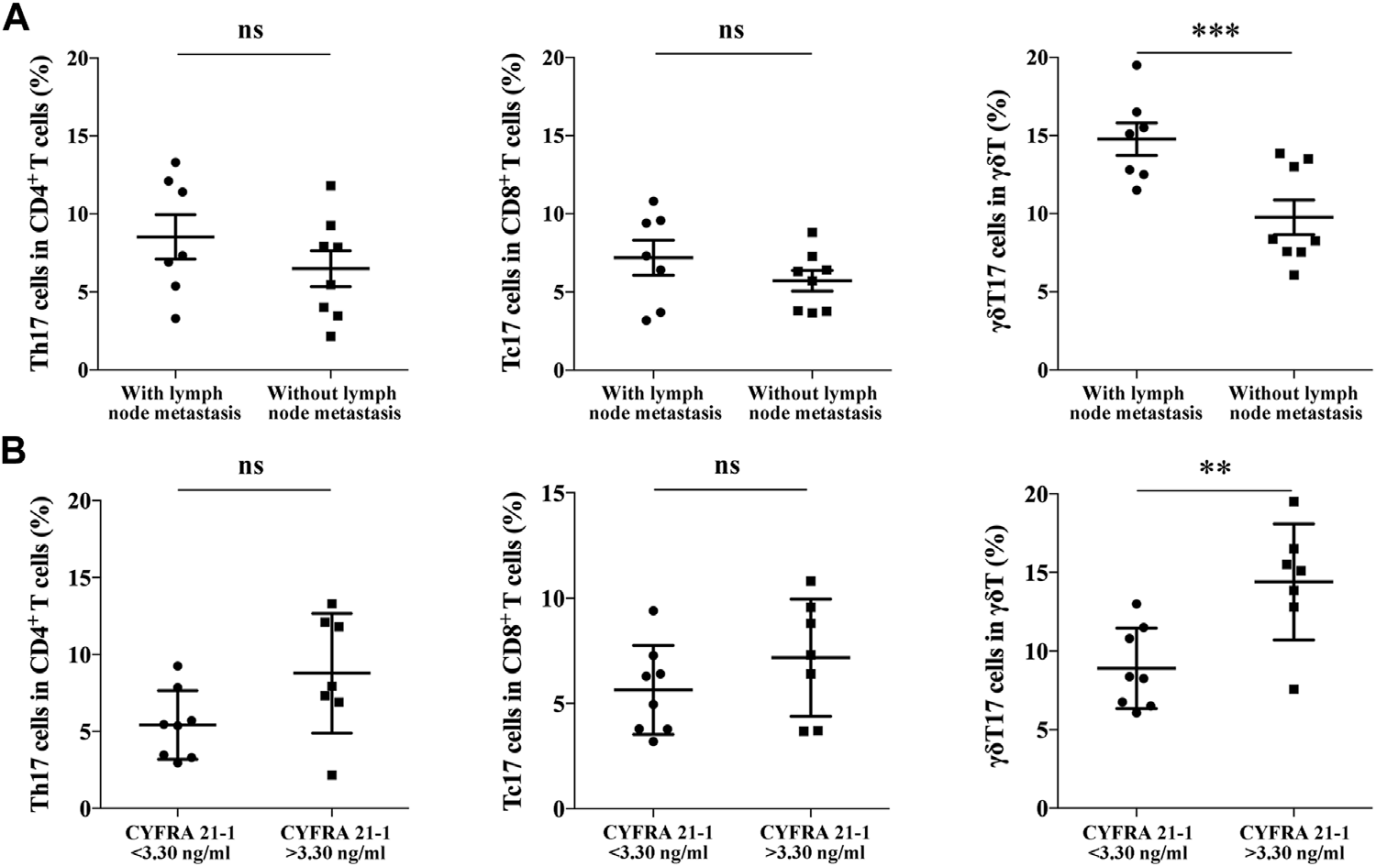
The correlation between Th17 cells, Tc17 cells, γδT17 cells in NSCLC specimens and clinical features of NSCLC patients. **(A)** The positive rate of Th17 cells (*n* = 15), Tc17 cells (*n* = 15) and γδT17 cells (*n* = 15) in TILs of NSCLC patients either with lymph node metastasis or not; **(B)** The positive rate of Th17 cells, Tc17 cells and γδT17 cells in TILs of NSCLC patients expressing high or low serum level of CYFRA 21-1. ***p < 0.001; **p < 0.01.

We further analyzed the correlation between IL-17^+^ γδT cells in TILs of NSCLC and clinicopathological features of NSCLC patients. As shown in Table 1, there was no significant correlation between different subsets of TILs and gender, age, smoking history, tumor size and CEA level. It is shown that the abundance of γδT17 cells was significantly higher in NSCLC patients with lymph node metastasis (14.77 ± 1.04% vs. 8.52 ± 0.92%, *p* < 0.001, Figure 3A), while no significant correlation was identified in Th17 cells and Tc17 cells. Moreover, a significantly higher abundance of γδT17 cells in TILs of NSCLC was detected in NSCLC patients expressing higher serum levels of CYFRA 21-1 (14.40 ± 1.39% vs. 8.91 ± 0.91%, *p* < 0.01, Figure 3B), indicating the involvement of γδT17 cells in the progression of NSCLC.

**TABLE 1:** Correlation of different IL-17A^+^ cell subsets with the clinicopathological characteristics of NSCLC.

Features	N	Subsets (*n* = 15)					
		Th17 (Mean ± SEM %)	*p* Value	Tc17 (Mean ± SEM %)	*p* Value	γδT17 (Mean ± SEM %)	*p* Value
Gender							
Male	8	7.18 ± 1.18	0.51	6.24 ± 0.71	0.87	10.02 ± 1.56	0.16
Female	7	8.56 ± 1.64		6.46 ± 1.08		13.06 ± 1.28	
Age							
<61	6	6.10 ± 1.40	0.61	5.86 ± 1.01	0.55	11.48 ± 2.29	0.97
≥61	9	7.30 ± 1.47		6.69 ± 0.86		11.41 ± 1.07	
Smoking							
Yes	5	8.73 ± 2.45	0.59	6.00 ± 1.00	0.71	10.44 ± 2.39	0.52
No	10	7.39 ± 1.19		6.54 ± 0.85		11.94 ± 1.14	
Tumor size (cm)							
<4	8	7.02 ± 1.41	0.89	6.96 ± 0.84	0.55	12.52 ± 1.41	0.30
≥4	7	7.29 ± 1.31		6.18 ± 0.95		10.21 ± 1.6	
Lymphatic metastasis							
Yes	7	8.89 ± 1.33	0.19	7.20 ± 1.11	0.23	14.77 ± 1.04	<0.001***
No	8	6.20 ± 1.39		5.62 ± 0.67		8.52 ± 0.92	
CYFRA 21-1 (ng/ml)							
<3.3	8	5.423 ± 0.79	0.06	5.639 ± 0.75	0.24	8.91 ± 0.91	<0.01**
>3.3	7	8.79 ± 1.47		7.18 ± 1.05		14.40 ± 1.39	
CEA (ng/ml)							
≤5	9	7.66 ± 1.20	0.79	6.77 ± 0.86	0.45	11.01 ± 0.97	0.64
>5	6	8.25 ± 1.91		5.74 ± 0.99		12.08 ± 2.36	

## Discussion

In the tumor microenvironment, the inflammatory responsive involving both inflammatory cells and inflammatory molecules remarkably influences chronic inflammation and tumor development. IL-17A is a powerful proinflammatory factor, which used to be considered as the product of Th17 cells, a subset of CD4^+^αβT cells. The secretion of IL-17A requires antigen selection and activation in the thymus, which is inconsistent with the large-scale production of IL-17A in the early stage of inflammatory response. A growing number of studies have later found that IL-17A can be produced by various types of innate immune cells like γδT cells and natural killer (NK) cells. Notably, γδT cells have been confirmed as the most important source of IL-17A in the early phase of immune response [5–7]. γδT cells mainly distribute in the first barrier of the body, which exert the role of toxic T cells by directly identifying antigens to invade surface of organisms. Umemura et al. [6] found that the administration of FasL-expressing tumor cells into mouse peritoneum remarkably increases the proinflammatory factors, including IL-17A. They reported that CD4^−^CD8^−^ cells, especially γδT cells are the main source of IL-17A, and the proportion of γδT17 cells in total γδT cells is much higher than that of Th17 cells in total αβT cells. γδT cells can rapidly produce IL-17A in the early stage of *Mycobacterium tuberculosis* infection, and last for 4 weeks, while CD4^+^T cells only produce IL-17 within the first 2 weeks of infection [8–10]. Therefore, we believed that γδT cells are the main source of IL-17A in the early stage of inflammatory response.

At present, the role of IL-17A in inflammatory response is controversial. γδT17 cells contribute to enhance the immunity to fight against infections of *Saphylococcus aureus*, *Mycobacterium tuberculosis*, and *Escherichia coli* by recruiting neutrophils and monocytes at infection sites [11–13]. In contrast, IL-17A can promote tumor development by promoting angiogenesis of endothelial cells and fibroblasts. Injection of NSCLC and cervical cancer cells overexpressing IL-17A is more likely to induce tumorigenicity in immunodeficient mice than those of controls [14, 15]. Production of serum IL-17 was significantly higher in the NSCLC group than in the control group and the expression levels were higher in patients with stage III and IV cancers than in patients at stage I or II [16]. Song L, et al. [17] found peripheral Th17 cells and γδT17 cells were significantly increased, whereas Tc17 cells were markedly decreased in patients with NSCLC compared with those in NC. Meanwhile Th17 cells and Tc17 cells were found associated with 5-year survival rate and TNM stage.

Howevever, the correlation between IL-17A and NSCLC has not been widely explored, and IL-17A level is usually measured in peripheral blood. We believed that IL-17A level in tumor specimens is more realistic to reflect the infiltration of inflammation in tumor sites, which is beneficial to identify the correlation between the microenvironment of NSCLC and inflammatory response. Cui K, et al. [18]found high frequency of γδT cells in patients with NSCLC and elevated γδT cells in tumor tissues were mainly IL-17A-releasing γδT17 cells. But they used paraffin-embedded tissues rather than fresh tissues.

In the present study, IL-17A was significantly upregulated in TILs of NSCLC specimens and the main source of it was γδT cells. Moreover, CYFRA 21-1 is a prominent marker for NSCLC with the outstanding sensitivity and specificity [19], and we found that the abundance of γδT17 cells was significantly higher in NSCLC patients expressing higher serum levels of CYFRA 21-1 and with lymph node metastasis, revealing that γδT17 cells were associated with the progression of NSCLC.

In summary, IL-17A was highly expressed in TILs of NSCLC and mainly from γδT cells, which promoted the development of NSCLC. IL-17A and γδT17 cells may be a promising target for immunoassay and immunotherapy of NSCLC. Due to the difficulty of obtaining fresh tissues, our sample size was small. Limited by this reason, our conclusions should be further validated in large-scale studies. In addition, the underlying mechanisms of IL-17A in mediating the microenvironment of NSCLC need to be explored in the future.

## Data Availability

The raw data supporting the conclusion of this article will be made available by the authors, without undue reservation.

## Footnotes

1 The authors have completed the TREND reporting checklist. The authors are accountable for all aspects of the work in ensuring that questions related to the accuracy or integrity of any part of the work are appropriately investigated and resolved.
